# Additive Manufacturing of Zirconia Ceramic and Its Application in Clinical Dentistry: A Review

**DOI:** 10.3390/dj9090104

**Published:** 2021-09-06

**Authors:** Leila Nasiry Khanlar, Alma Salazar Rios, Ali Tahmaseb, Amirali Zandinejad

**Affiliations:** 1 Department of Cariology and Operative Dentistry, Graduate School of Medical and Dental Sciences, Tokyo Medical and Dental University, 1-5-45, Yushima, Bunkyo-ku, Tokyo 113-8510, Japan; 2 College of Dentistry, Texas A&M University, Dallas, TX 75246, USA; als-81@tamu.edu (A.S.R.); azandinejad@tamu.edu (A.Z.); 3 Department of Oral Maxillofacial Surgery Erasmus Medical Centre, 3015 Rotterdam, The Netherlands; ali@tahmaseb.eu

**Keywords:** additive manufacturing, zirconia ceramic, dental applications

## Abstract

Additive manufacturing (AM) has many advantages and became a valid manufacturing technique for polymers and metals in dentistry. However, its application for dental ceramics is still in process. Among dental ceramics, zirconia is becoming popular and widely used in dentistry mainly due to its outstanding properties. Although subtractive technology or milling is the state of art for manufacturing zirconia restorations but still has shortcomings. Utilizing AM in fabricating ceramics restorations is a new topic for many researchers and companies across the globe and a good understanding of AM of zirconia is essential for dental professional. Therefore, the aim of this narrative review is to illustrate different AM technologies available for processing zirconia and discus their advantages and future potential. A comprehensive literature review was completed to summarize different AM technologies that are available to fabricate zirconia and their clinical application is reported. The results show a promising outcome for utilizing AM of zirconia in restorative, implant and regenerative dentistry. However further improvements and validation is necessary to approve its clinical application.

## 1. Introduction

The unique properties of zirconia ceramic as restorative dental material, along with the growing demands from patients for aesthetic and metal-free restorations, have attracted the attention of the dental profession. Currently, zirconia restorations are widely used in dentistry due to their biocompatibility, chemical stability, outstanding mechanical properties, and optical characteristics [1]. Full-contour zirconia restorations have been the first choice for restoring posterior teeth in the United States [2]. Furthermore, with increased translucency and advancements in coloring procedures, monolithic zirconia restorations are becoming more popular in clinical practice. Due to their mechanical properties, zirconia restorations also present other advantages such as minimal tooth preparation requirements and eliminating the risk of veneer chipping for porcelain fused to zirconia restorations [3].

Since its introduction in dentistry in the 1970′s [4], subtractive computer-aided manufacturing (CAM) technology such as milling, has emerged as the main approach for producing zirconia restorations. Restorations can be created using two methods: “soft machining” or “hard machining” of fully sintered blocks [5]. The soft machining method, which is based on the milling of pre-sintered blocks followed by sintering, is the most widely used manufacturing technique for yttrium-stabilized zirconia (YSZ) [5,6]. This leads to a fairly even and homogenous distribution of the components within the block and a very small pore size (20–30 nm) [5]. Finally, the framework is sintered at high temperatures and achieves its ultimate mechanical properties by undergoing a linear volumetric shrinkage of approximately 25%, regaining its correct dimensions. Such processing creates very stable cores with a large quantity of tetragonal zirconia and surfaces that are almost free of monoclinic phase unless grinding adjustments or sandblasting are needed [5,7]. In contrast, the hard machining method involves the milling of dental restorations from fully sintered blocks, which eliminates the possibility of shrinkage of the final prosthesis. This can be advantageous but can also lead to a phase transformation from tetragonal to monoclinic on the surface associated with accelerating low temperature degradation (LTD) and causing microcracks, which has a detrimental impact on the longevity of final restorations [5,6,8,9]. On the other hand, the hard milling technique requires the use of special milling machines which are able to mill sintered ceramics as well as cutting tools that must be extremely robust and resistant to wear [6,10,11]. Although the subtractive method is a well-established technology with the advantages of using homogenous materials independent of operating conditions, but it also has some drawbacks which results in large material loss and high cost as well as the accuracy of the procedure which can be limited by the factors such as object’s complexity and the tooling machinery’s size and material properties [12,13].

Additive manufacturing technology (AM) is an alternative to address the drawbacks of subtractive manufacturing in the CAM step of the dental digital workflow, also known as 3D printing, which is capable of fabricating devices by layering materials using a computer-generated design file; standard tessellation language (STL). Even though AM has yet to be developed, it enables the production of ceramic parts with complex geometries, high precision, and low cost [4,14,15].

The American Society for Testing and Materials (ASTM) divides AM technologies into seven classifications to construct a product layer by layer: stereolithography (SLA), material jetting (MJ), material extrusion (ME), binder jetting (BJ), powder base fusion (PBF), sheet lamination (SL), and direct energy deposition (DEP), as all can be used to form ceramic components [16,17,18].

Various AM techniques have been utilized to manufacture zirconia objects including vat photopolymerization (stereo-lithography and direct light processing), selective laser sintering, selective laser melting, ink-jet printing, fused deposition modeling, direct energy deposition, sheet lamination, and binder jetting [19]. Although this review will cover all available techniques for AM zirconia, it will primarily examine the most common ones for additively manufacture zirconia.

## 2. Additive Manufacturing Techniques for Zirconia Ceramic

### 2.1. Vat Photopolymerization

Vat photopolymerization technologies are defined as “additive manufacturing processes in which liquid photopolymer in a vat is selectively cured by light-activated polymerization” [19]. This involves techniques such as stereolithography (SLA) and digital light processing (DLP).

#### 2.1.1. Stereolithography (SLA) 

**Figure 1:**
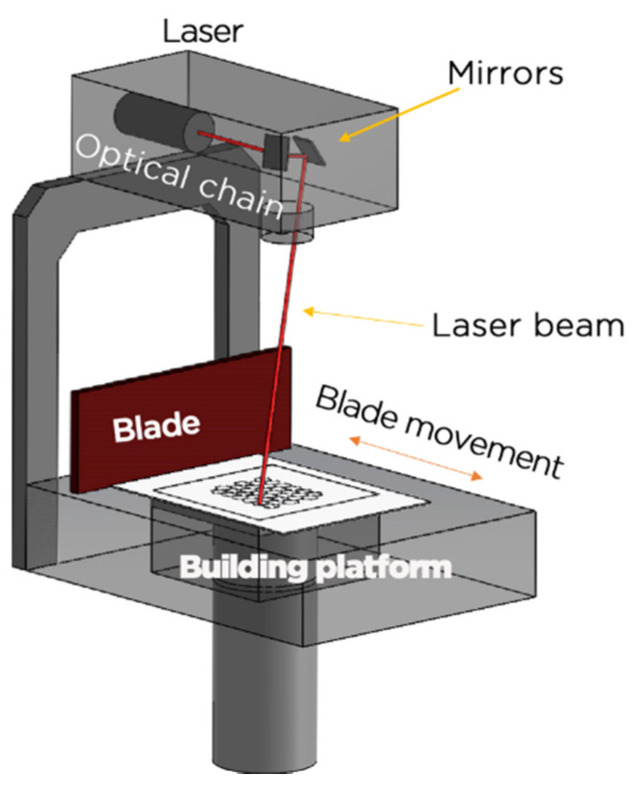
Schematic illustrations of the SLA apparatus (courtesy of 3Dceram).

SLA was the first AM technology to be utilized in the medical field and utilized to fabricate surgical models for alloplastic implant surgery in1994 [20]. SLA is a process in which a light spectrum either laser or light-emitting diode (LED), is used in vat polymerization printing to construct parts one layer at a time in a vat containing light-cured photopolymer resin combined with ceramic powder [21,22]. The light passes through each layer of the liquid resin’s surface. The construction plate then drops, allowing another layer of resin to spread across the surface and thereby repeating the process. Figure 1 shows a schematic diagram of SLA apparatus. The curing depth is a crucial key to determining the formability and accuracy of the technique. This technique is distinguished by a high degree of accuracy and surface quality in addition to its capacity to generate complex shapes [15] without the need for the use of a high-energy laser beam. As a result of these characteristics, SLA has become one of the most essential and widely used AM technologies for manufacturing of zirconia devices [23,24]. The SLA of ceramics begins with the incorporation of fine ceramic particles as small as micro/nanometers into the photocurable solution [25,26]. After the liquid has been thoroughly dispersed in the medium with the help of necessary surfactants and additives, it creates a ceramic suspension. Similarly, because ceramic particles are inert to light emission, polymerization occurs exclusively in the organic monomer phase when exposed to light. The ceramic particles are then uniformly enveloped by a cross-linked organic network that is polymerized to form the pre-designed shape of each layer until the full 3D ceramic item is constructed. The green components must go through additional processing, often pyrolysis to remove organic components (like burn out) followed by high-temperature sintering to achieve the desired density [25,27]. The ceramic suspensions play a critical role in the process and are primarily controlled by light adsorption and rheology. To manufacture a zirconia item that is free of defects and performs well, a photosensitive suspension with a high solid loading, low viscosity, and homogeneous dispersion is required [25]. Ceramic resins for SLA should have a viscosity of less than 3000 cps for self-leveling during the 3D printing process, although the viscosity of the ceramic suspension increases significantly with increasing ceramic content. Despite the fact that ceramic resins should have as high a solid loading as feasible in order to manufacture ceramic 3D-printed objects with adequate densification, and also as reported the solid loading of the ceramic suspension must be greater than 40% to avoid defects during debinding and sintering, but most ceramic resins have a loading of less than 40% by volume which can lead in zirconia objects with defects, low density, and high shrinkage [21,23,25,28]. This is usually challenging since lower ceramic loading is needed to reduce viscosity and prevent potential solid content segregation, on the other hand, to obtain higher density, less shrinkage, and therefore higher mechanical properties, higher volume percentage of ceramic particles is advantageous. Zhang et al. [29] recently developed a high loading (55 vol%) photosensitive ZrO_2_ suspension, which provided new possibilities for fabricating high performance ZrO_2_ objects by SLA.

Another major issue to address is the significant effect of light scattering caused by the ceramic particles added to the suspension, even if the particles themselves are transparent to irradiation. The light penetration into the suspension is deteriorated by this scattering and it can impact the dimensional accuracy [30]. The ceramic particle size, volume percentage, materials’ reflective index, and light exposure energy may affect the curing depth and hence the layer thickness [25]. The difference in refractive index between the photopolymerizable liquid and the zirconia particles is very significant. For zirconia particles with higher refractive index when exposed to the UV light, more UV radiation may be absorbed and resulting in shorter depth of curing and greatly reduce the photopolymerization reaction [31]. Following the manufacturing of the green pieces, they are conducted to debinding to remove the remaining organic resins. Several factors influence debinding behavior including resin composition, solid loading, and component shape [32]. Debinding can make cracks which is probably due to internal stresses resulting from thermally-initiated polymerization [33]. Sintering is the final procedure in which the pores between the particles are removed to obtain the fully dense zirconia objects with high performance. Sintered zirconia densification and strength are affected by sintering temperature and gradient [34]. 

Among the different AM technologies, stereolithography (SLA) is the most preferred for dental applications because it provides the highest accuracy, resolution, and a flawless surface finish [35]. There are a few novel studies which investigated the physical and mechanical properties of AM zirconia which are required for dental applications. Revilla-León et al. [36] examined the flexural strength SLA additively manufactured bar-shaped zirconia applications and the effect of aging simulation on their flexural strength and compared to milled zirconia. The results indicated less flexural strength for AM zirconia than milled zirconia. Moreover, mastication simulating aging had a significant decrease in both AM and milled groups in terms of flexural strength and fracture resistance. In another study, Revilla-León et al. [37] investigated the manufacturing accuracy and volumetric changes of SLA additively manufactured zirconia specimens with porosities of 0%, 20%, and 40%. A bar-shaped digital design and a photopolymerizable ceramic suspension was used in the SLA technology. To obtain various porosities, the ZrO_2_ sintering methods differed between groups, with 1450 °C temperature for 0% group, and between 1450 °C and 1225 °C for 20% and 40%. The dimensions of specimens measured by digital caliper and the volume shrinkage percentage of fabrication was computed by comparing the digital design of the bar with dimensions of final AM specimens. Although none of the groups examined were able to fully mimic the specimens’ virtual design, the AM zirconia with a porosity of 40% had the highest fabricating accuracy and the smallest volume change after manufacturing, followed by the 20% porosity and 0% porosity groups. Nakai et al. [38] evaluated the crystallography, microstructure and flexural strength square-shaped SLA additively manufactured zirconia-based ceramics and compared with milled zirconia. AM 3Y-TZP; LithaCon 3Y 230 (Lithoz) with printing orientation 90-degree and 3D Mix zirconia (3DCeram Sinto) with 0-degree printing orientation and one alumina-toughened zirconia (ATZ) with 0-degree were compared with milled 3Y-TZP zirconia. The results showed that the phase composition and residual porosity of three additively manufactured zirconia ceramics were similar to that of milling zirconia. The strength of additively manufactured 3Y-TZP were comparable to that of milled 3Y-TZP, according to biaxial flexural strength tests. Between AM groups, alumina-toughened zirconia (ATZ), showed the highest strength followed by AM zirconia with SLA technology printed in 0-degree angulation (Perpendicular to load direction), while DLP AM zirconia with 90-degree printing angulation (parallel to load direction). 

#### 2.1.2. Direct Light Processing (DLP)

Digital light processing (DLP) is based on vat-polymerization technology and is similar to SLA and can be classified into same category but it differs in terms of the light source used [39]. An arc light or microscopic mirrors spread out in a matrix on a semiconductor chip provide the image for DLP. This matrix is referred to as a digital micromirror device (DMD). A DMD is a chip that is made up of a rectangular array of thousands of microscopic mirrors that correlate to the pixels in the image to be exhibited. Each mirror demonstrates one or more pixels in the projected image; thus, the number of mirrors correlates to the projected image’s resolution [40,41]. Because of ultra-fast light shifting and integral projecting, DLP AM has higher printing speed than SLA technique. Besides that, higher resolution can be achieved by using several micromirrors [42,43]. In contrast to SLA, where the laser beam moves across the layer surface, causing localized polymerization of the photosensitive resin in the illuminated field, DLP cures the entire material portion in the x/y space simultaneously by a single projection of the entire layer through the light projector [44].

DLP can construct the parts in either a bottom-up or top-down layout. In the bottom-up set-up, an object is created upside down on the building platform and dipped in a thin slurry layer deposited on a glass plate rather than entirely immersed in the liquid resin. The advantages of this method are the little quantity of slurry required for producing the parts and less expensive than the top-down printers [40,45]. DLP systems were designed to manufacture complicated ceramics parts which need a high degree of details and accuracy [41]. 

### 2.2. Selective Laser Sintering (SLS) 

Selective laser sintering (SLS) is a powder-based additive manufacturing (AM) technology that uses powder beds containing loose ceramic particles as feedstock to construct three-dimensional objects [46]. As the name implies, a high-power laser beam is used in SLS method to selectively irradiate the surface of the desired powder bed. The powder is then heated, and sintering occurs for mass joining. Following that, a new coating of powder is applied to the prior surface in preparation for the next round of heating and joining. The technique is then repeated layer by layer until the planned 3D part is completed [46,47]. The SLS process does not require any additional support structures because overhangs and undercuts are always encompassed by the loose powder bed [47].

The SLS process can be classified into two types to manufacture ceramics: direct and indirect. In indirect SLS, ceramic powders are combined with a polymer binder and the scanning laser melts the binder, which bonds the ceramic particles together. The binder is removed and portions are densified during the subsequent debinding and sintering [48]. In direct SLS, no polymer binder is employed, and the laser beam directly sinters the ceramic particles. If the delivered laser energy density is fairly high, the interaction of the laser with the ceramic particles will induce the particles to bond together. Thus, no further de-binding or furnace sintering is required [49].

Materials with a high melting point like zirconia ceramics are particularly difficult for the SLS process as their densification requires high temperatures and a lengthy time to obtain acceptable densities. Another common challenge with these ceramics is that thermal stresses during SLS might trigger crack formation in sintered objects [50]. It was found that colloidal techniques such as slurry deposition by blade coating [51] or spray deposition [52] could be used to improve the density of the powder layer. Aside from changing the powder shape, mixing with another low melting point compound, such as a polymer binder, could be utilized to improve liquid phase sintering in the laser process and, as a result, enhance ceramic density [48,53]. Moreover, it was reported that powder bed preheating can lower thermal stresses and consequently crack formation in SLS ceramic products [48,49]. The notable shrinkage of ceramics and the high porosity in the finished pieces are the two main issues in the SLS of ceramics [14]. On the other hand, laser power and scan speed can impact the surface roughness as less laser power and high-speed scan can result in decreasing the roughness. It was also mentioned that the strength of the pieces would deteriorate in the same manner [51]. A recent study showed that the application of ultrasonic vibration aided in the decrease of grain size and indeed increasing the wear resistance, microhardness, and compressive properties [54]. Therefore, optimizing the powder properties and laser processing parameters has been noticed to eliminate cracks and increase density. 

### 2.3. Selective Laser Melting (SLM) 

Selective laser melting (SLM) is another powder-based AM technique which works similarly to SLS except that it is a one-step bed fusion by full melting that uses laser sources with substantially higher energy densities and does not require any secondary low-melting binder or post-treatment [55]. SLM is one of the fastest expanding AM technologies, especially to create solid pieces from metal powders. SLM of ceramics is more challenging than SLM of metals. They have a lower density than metals. As a result, ceramic powders have poor flowability, making it difficult to spread a thin coating of ceramic powders evenly on the building platform [56]. However, spray-drying can increase the flowability of an alumina-zirconia powder mixture, allowing for the deposition of a thin layer on the platform [56].

Ceramics, particularly zirconia also have high melting temperatures, extremely low thermal conductivity, and limited ductility. The thermal stress caused by extremely short laser–powder interaction durations and large amounts of energy required for melting, meaning the abrupt heating and cooling rates with each laser scan, is one of the most severe difficulties resulting from the SLM method. As a result of the poor thermal shock resistance of ceramic (zirconia) materials, cracks are more prone to emerge in sintered parts as a result of such thermal stresses combined with the limited ductility of ceramic materials [56,57]. Only a few investigations have been conducted on the selective laser complete melting of zirconia. They demonstrated the use of SLM to manufacture zirconia objects while flaws such as cracks and large open pores or lower density of the final product were observed [58,59]. Recent studies showed that preheating the ceramic powder bed was more beneficial in reducing such difficulties caused by thermal gradient. High-temperature preheating of the zirconia ceramic powder appears to be the most effective approach for minimizing cracks during the SLM process [60]. Moreover, it was found more advantageous to pre-heat the ceramic powder for aiding in the homogeneity of energy distribution [61,62] and having a significant impact on density of materials [60]. The specimen’s crystal structure after preheating was predominantly tetragonal. It was also proved that as the preheating temperature increased, the tetragonal crystal became more visible [60]. Furthermore, the preheating procedure may assist in the improvement of mechanical performance due to inhibited crack formation [61,63].

### 2.4. Direct Inkjet Printing (DIP) 

Direct Inkjet printing as a material jetting technology is a well-known method which allows for the manufacturing of ceramic objects with full density, high accuracy, complicated shapes, and minimal material usage at a low cost; conversely to other methods which construct porous ceramic structures [64,65]. A suspension comprised of ceramic powder particles is deposited (directly) from a print nozzle during direct inkjet printing (DIP). Individual droplets of the suspension are selectively deposited onto a substrate by the print nozzle. When the droplets come into contact they experience a phase transition, resulting in the formation of a solid portion. DIP ceramic manufacturing method works by rapidly heating the nozzle to vaporize the ceramic ink in the capillary at the bottom of the nozzle and generate bubbles that rapidly expand. The bubble would develop to a crucial size, breaking the surface tension of the ink and causing it to discharge from the top of the nozzle. The ceramic ink is drawn according to the computer-pre-modeled data, and the layer is superimposed to create 3D ceramic parts [66,67,68]. 

The efficacy of Inkjet printing of ceramics is highly dependent on key elements including ceramic powder and ink composition as well as their rheological features like dispersity, viscosity, and surface tension [14]. The performance of the zirconia pieces might be improved by properly designing the ink characteristics and ejection parameters such as the discharge rate and nozzle speed as well as the distance between the nozzle and previously deposited layers [64,69,70]. Another concern with ceramic Inkjet printing is the coffee staining effect, which can occur during the drying process of printed patterns owing to convective macroscopic flow into the contact line. This phenomenon exhibits itself as the separation of solid particles from the center to the edge of the printed patterns on the substrate and resulted in defects in the printed objects [71,72]. The coffee staining behavior of printed drops was studied on a variety of substrates, such as glass microscope slides, epoxy resin, and preprinted and dried ZrO_2_ powder layers [73]. On solid substrates, coffee staining can be eliminated by incorporating 10% poly (ethylene glycol) (PEG) into the original ink. Coffee stains were fully eradicated at room temperature (25 °C) using the modified ink. Nevertheless, coffee staining was found again at temperatures over 35 °C, which can be described by diffusion lowering the concentration gradients that cause Marangoni flow. Coffee staining observed at room temperature when the PEG-modified ink was printed over a layer of dried ceramic powder, which is associated to the drops drying by the draining of fluid into the dried powder bed. Moreover, it has been shown that the use of a high vapor pressure solvent like 50 vol% isopropyl alcohol for faster drying represented a rapid increase in the viscosity of the ceramic ink following deposition and a totally suppressed coffee strain effect [74].

### 2.5. Binder Jetting

Binder jetting is a method of additive manufacturing which powder particles are bonded together using a liquid binding agent that is selectively deposited. The bonding of material layers results in the formation of an object. Following that, unbound powder is removed from the green part and appropriate post-processing, such as sintering, is performed [75,76]. Thermal inputs are eliminated in the binder jetting process; thus, it could prevent the formation of residual stresses in the pieces, which is a typical problem with other additive manufacturing technologies [77]. Different factors influence the accuracy and strength of binder jetting printed objects, including the powder materials used, the binder (binder amount, drying power level and period), the orientation of the parts, geometric characteristics, and post-processing [75,78].

### 2.6. Fused Deposition Modeling (FDM)

Material extrusion, also known as fused deposition modeling, in where the ceramic material is heated over its melting temperature and extruding through a nozzle. Then, one layer at a time, it is deposited. The nozzle moves horizontally and the platform travels vertically to allow for the addition to each subsequent layer. The printed part is conducted for debinding and sintering to obtain densification [19,79]. The layer thickness, which is controlled by the nozzle size, determines the object’s vertical dimensional resolution. Flexible ceramic filament is difficult to make since the mixture of polymer binder and ceramic powder look brittle [80]. The process factors, such as rod width (fused ceramic/polymer filament), layer thickness, printing orientation, and raster angle, have a significant impact on the quality of the printed objects, including homogeneity, surface roughness, dimensional accuracy, and mechanical qualities. For FDM fabricated applications, surface roughness is the main concern [14].

### 2.7. Direct Ink Writing (DIW)

Direct ink writing (DIW), also known as robocasting, was initially designed to process concentrated materials with limited organic component, such as ceramic slurries [14]. The ceramic suspension with a high solid loading is built up in the DIW process by moving nozzles to directly “write” the designed shape layer by layer until the part is complete. It is usually followed by debinding and sintering to remove any organics from the component [22,32]. One of the major issues for DIW of ZrO_2_ is the development of improved ZrO_2_ inks capable of generating complex 3D structures with micro- and nano-scaled resolution and large span capability [32]. The direct write printing technology provides an advantage in structural design and precise control over the porosity, which will enable prospective applications in scaffold production and tissue engineering [22,81].

## 3. Dental Applications of AM Zirconia Ceramics

**Figure 2:**
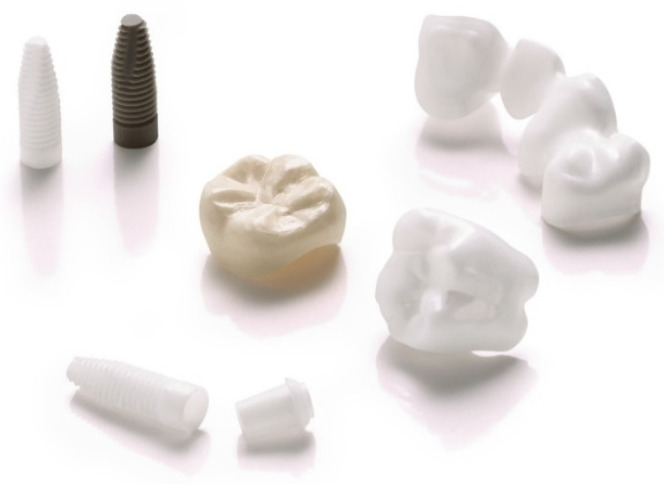
3D-printed dental medical devices made on a CeraFab System S65 Medical: Dental implants (left top), zirconia bridge (3Y-TZP, right top), molar crown precolored (center), molar crown uncolored (right bottom), zirconia implant and abutment (center bottom). (Courtesy of Lithoz GmbH, Vienna, Austria).

Despite its numerous advantages, AM has yet to be certified as a fabrication technology for zirconia ceramic restorations. Although there has been limited research on the 3D printing of dental zirconia ceramics, current studies show a promising future for AM zirconia ceramics in dental applications. So far, some research has concentrated on additive manufacturing approaches for fabricating zirconia dental applications with commercially available systems which in this review paper is classified into three categories: restorative applications, zirconia implants, and bone regeneration. Table 1 illustrates the summary of the dental research related to AM zirconia dental applications. Figure 2 shows different prostheses has been manufactured by additive manufacturing. 

Many recent AM zirconia ceramics research attempts have focused on the use of stereolithography-based technologies, as one of the most promising techniques. Recently, a few AM manufacturers which are using stereolithography-based technologies are available and can print zirconia could be prospective suppliers for zirconia printing for dental applications. Table 2 shows all of the 3D printers available for manufacturing zirconia ceramics.

### 3.1. AM Zirconia Restorative Applications

Ebert et al. [65] employed direct ink-jet printing as a promising technology for fabricating zirconia dental restorations using a modified ink-jet printer. The researchers were able to print zirconia with a layer thickness of 100 μm and the similar size and shape of a posterior crown using a 27 vol% zirconia based ceramic suspension. This approach yielded zirconia with a fired specimen’s density of 96.9%, characteristic strength of 763 MPa and a fracture toughness of 6.7 MPa. Furthermore, the well-designed drying and cleaning procedure resulted in the formation of crack-free three-dimensional pieces of the size presented. However, the printed and fired specimens’ microstructures were not fully devoid of process-related flaws, which were caused by single clogged nozzles that dried out or became blocked by agglomerates during the printing process.

Özkol et al. [70] developed aqueous inks of 3Y-TZP and carbon for the direct inkjet fabrication of a zirconia dental bridge framework. Finite element analysis (FEA) to determine the stress distribution in the framework and a four-point bending for analyzing the maximum tensile strength were employed by researchers. Ceramic and supporting inks were developed and printed separately. The ceramic ink was an aqueous dispersion of 3Y-TZP particles with submicron sizes. A dispersant based on carboxylic acid was utilized. To make the ceramic ink, the suspension was diluted to 27 vol% solids content by adding water, dispersants, and humectants to reduce viscosity and optimize drop ejection behavior. Using the described AM technique, the authors got ceramic components with a smooth surface and no stair-step effect, drying, or sintering cracks, as well as a relative density more than 96% of the theoretical density. The FEA results for all different loading cases revealed hot spots on the interdental connectors’ bottom marginal area. The estimated maximum tensile stress values ranged between 250 and 350 MPa. Regarding the four-point bending test, printed specimens had a high flexural strength of about 843 MPa.

Lian et al. [23] investigated the use of zirconia as an AM material for complex dental bridges using SLA technology. The authors utilized 40% vol% aqueous zirconia suspension and a scanning speed 1200 mm/s. The green body has a porosity rate of 23.46%. The apparent porosity of final zirconia objects decreased during sintering and density of solid sharply increased. They were able to fabricate zirconia ceramic bridges with relative density of 98.58%, Vicker’s hardness of 1398 HV, surface roughness (Ra) of 2.06 μm, isotropic shrinkage of 20 to 30 vol%, and flexural strength of about 200 MPa, although internal defects were detected by SEM analysis in the final objects.

Wang et al. [85] investigated the 3D trueness stereolithography (SLA) additively manufactured zirconia crowns and compared with crowns fabricated by subtractive milling technology. The trueness of external, intaglio, and marginal area of the 3D-printed crowns were comparable with the trueness of the milling crowns. Rather than directly testing the fit of the crowns (as with replica technique or sectioning multiple dies), they examined the point-to-point discrepancies between the scan data and the corresponding CAD model data by color mapping to analysis the trueness of the crown-manufacturing process.

Li et al. [86] assessed the physical and mechanical properties of stereolithography (SLA) additively manufactured zirconia crowns, as well as the internal and marginal adaptation of zirconia ceramic dental crowns. The strength of the SLA-manufactured zirconia crowns in this investigation was sufficient to build dental crowns, flexural strength of 812 ± 128 MPa and a Weibull modulus of 7.44. In this investigation, a 45-vol% zirconia suspension, a modest layer thickness, suitable laser intensity, and a prolonged exposure duration were used to obtain reliable mechanical strength. Furthermore, a low heating rate was used. Nevertheless, the internal and marginal adaptations were not suitable for clinical application and that could be due to light scattering and anisotropic sintering shrinkage.

**Figure 3:**
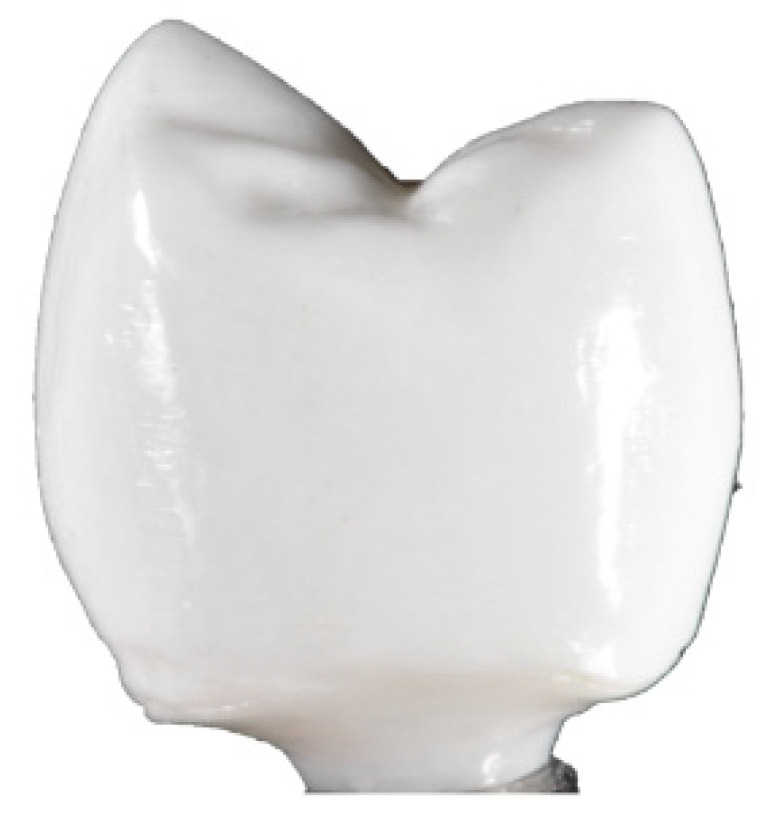
Stereolithography (SLA) additive manufacturing (AM) zirconia crown with buccal marginal defects.

Revilla-León et al. [87] compared the marginal and internal discrepancies of milled and stereolithography (SLA) additively manufactured zirconia crowns by using the silicone replica technique. For AM groups, they used AM anatomic contour zirconia and AM splinted zirconia. They reported a significant lower marginal and internal discrepancies in the milled method. Moreover, the milled and splinted groups showed marginal and internal discrepancies that were clinically acceptable, whereas the anatomic contour zirconia crown had clinically inappropriate marginal and internal discrepancies (Figure 3).

Ioannidis et al. [88] evaluated the load-bearing capacity of additively manufactured zirconia ultra-thin occlusal veneers on molars and compared to milled zirconia and heat-pressed lithium-disilicate. Occlusal veneers with 0.5 mm thickness on molars digitally were designed. To fabricate AM zirconia veneer, lithography-based ceramic manufacturing (LCM) process (similar to DLP) with a photopolymerizable monomer mixture containing different ceramic powders with 40–60 vol% concentration was used. The restorations were cemented to tooth structure using an adopted bonding process. After chewing simulator as aging process, load-bearing capacity was measured. The researchers found that while significant differences were observed in three materials, all of their load-bearing capacities were surpassed clinically expected average bite forces. As a result, they concluded that for fabricating the ultra-thin occlusal veneers to restore occlusal tooth wear, AM zirconia, also milled and heat-pressed lithium disilicate, can be considered as restorative materials.

In a recent study, Wang et al. [89] assessed the dimensional accuracy and clinical adaptation of AM stereolithography fabricated ceramic crowns. Dimensional accuracy was determined by superimposing the digital casts with the reference model and to assess clinical adaption, the silicone replica method was used. The internal space for the cement was established at 30 µm, and a crown was built using typical occlusal morphology. They compared two different SLA systems, CeraFab7500 for Alumina (CF) and CSL150 for zirconia (CL) materials both with the layer thickness of 25 mm and compared to zirconia milled crown (XM). The results showed the dimensional accuracy was higher in CF (41 ± 11 µm) than in CL (65 ± 6 µm) or XM (72 ± 13 µm). The ceramic slurries, light sources, and polymerization techniques used in two SLA systems differed, resulting in dimensional variances. There was no significant difference between the AM zirconia crown and milled zirconia crown. When compared to CF and CL, XM demonstrated significantly superior adaptation in the marginal and occlusal areas but weaker adaptation in the axial area. Only in the axial and occlusal areas were significant differences found between CF and CL. They concluded that both systems manufactured ceramic crowns with high dimensional accuracy and marginal adaptability within clinically acceptable limits.

Li et al. [90] evaluated effectiveness of SLA manufactured zirconia crown with three finishing line designs including chamfer, rounded shoulder, and knife-edge and compared them to milled crowns. Manufacturing accuracy was determined using 3D deviation analysis and margin quality was assessed using microscopes. Three digital abutment models with a 0.5 mm depth chamfer, 0.5 mm depth rounded shoulder, and knife-edge finishing line were constructed. The abutments had a 1.0–1.5 mm occlusal reduction and a 6–10° taper. The results indicated that the finish line design but not the fabrication technique had a significant impact on RMS value. On the other hand, the AM and milling crowns varied in terms of error distributions in the external surfaces using color difference mapping. AM crowns had rounded line angle margins and were free of tiny faults, while crowns in the milling group had sharp line angle margins and discrete chippings. Although AM crowns indicated comparable accuracy to milled crowns, in both AM and milled crowns, knife-edged crowns were susceptible to large marginal chipping.

Additive manufacturing has been shown to generate both fully sintered (solid) and partially sintered (more porous) structures by varying manufacturing conditions. As a result, introducing porosities may change mechanical properties, which could be useful in mimicking the mechanical properties of enamel and dentin and enabling the fabrication of functionally graded dental restorations [91].

### 3.2. AM Zirconia in Implant Dentistry

Cheng et al. [82] investigated mechanical characteristics and microstructure of sintered implants fabricated by 3-dimensional slurry printing (3DSP) system. To make the slurry, zirconia-yttrium (3 mol%) ceramic powder with a particle size of 1 μm was mixed with a photopolymerizable resin in a 13:5 weight ratio. The binder was a photopolymer consisting of triethylene glycol dimethacrylate and urethane dimethacrylate in a 1:3 weight ratio. A two-stage sintering procedure was utilized. The green body and sintered specimens have flexural strengths of 20.41 ± 3.8 and 632.1 ± 72.5 MPa, respectively. The Vickers hardness test also revealed that the green body had a low hardness (0.12 GPa) when compared to the sintered part (14.72 GPa). An optimized model of the dental implant with the least amount of micromotion was obtained. However, the accuracy of final parts required to be enhanced.

Moin et al. [83] investigated the possibility of constructing three-dimensional (3D)-printed zirconia root analogue implants (RAI) using digital light processing (DLP) technology from a cone-beam computed tomography (CBCT) dataset. The optical surface model of the original tooth and the CAD model were superimposed to verify the accuracy of the printed zirconia RAI. The photopolymer resin that was used to create a solidified ceramic object was a dispersion of a commercial ceramic powder in a liquid polyacrylate solution. Results showed the printed RAI had a 6.67% more surface area than the original tooth, and 46.38% of the RAI deviated more than 0.1 mm from the original tooth. In comparison the printed customized implant with the CAD model measurements revealed a greater divergence for surface area higher change percentage threshold exceeding for 0.1 mm, which indicated a larger copy. This demonstrates that the current level of DLP technology delivers less accurate zirconia ceramic models than SLM additive manufacturing technology for titanium models. According to their results, the 3D printed RAI obtained high dimensional accuracy and the authors concluded the feasibility of manufacturing one-piece zirconia (root analogue) implant (RAI) using the DLP technology.

Osman et al. [84] evaluated the dimensional accuracy and surface topography of a custom-designed, 3D-printed zirconia dental implant, as well as the flexural strength of printed zirconia discs with three different printing orientations (0, 45, 90 degree) using digital light processing (DLP). It was demonstrated that the DLP technology could print customized zirconia dental implants with enough dimensional accuracy with a root mean square (RMSE) value of 0.1 mm. Cracks, microporosities, and interconnecting pores ranging in size from 196 nm to 3.3 µm were detected using SEM. The mean Ra parameter (arithmetic mean roughness) was 1.59 ± 0.41 µm, while the Rq parameter (root mean squared roughness) was 1.94 ± 0.47 µm. The flexure strength was comparable to conventionally produced zirconia ceramics and exhibited values around 943 MPa. The highest flexure strength values were obtained with a 0-degree vertical printing orientation, while the lowest values were obtained with a 45-degree. The Weibull analysis found that 0-degree printed specimens had a statistically significant greater characteristic strength (1006.6 MPa) than the other two groups, but there was no significant difference between 45-degree (892.2 MPa) and 90-degree (866.7 MPa) printing orientations. The authors reported acceptable flexural strength for AM manufactured zirconia.

**Figure 4:**
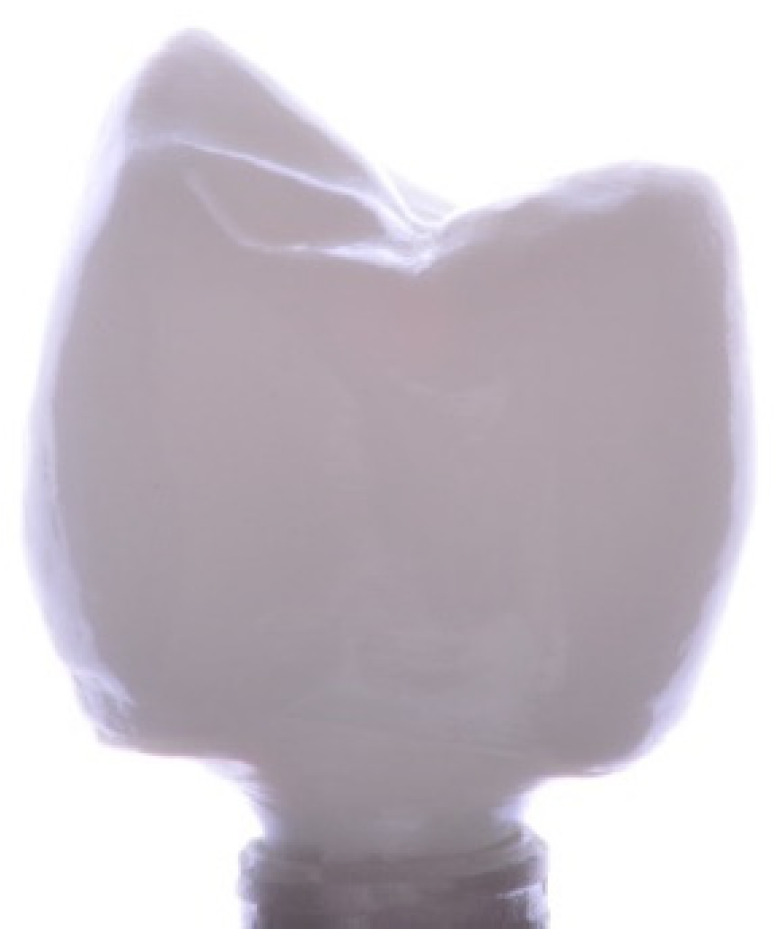
Stereolithography (SLA) additive manufacturing (AM) of implant supported zirconia crown after cementation.

The fracture resistance of implant-supported milled zirconia, milled lithium disilicate, and stereolithography (SLA) additively manufactured zirconia crowns were compared by Zandinejad et al. [18]. Zirconia implant abutments were designed with a chamfer finish line and having 6 mm buccal and lingual wall height and 4 mm proximal wall height. The abutments were prepared with a 10° to 12° total convergence angle and a 1 mm chamfer margin. The crowns were cemented to implant-supported zirconia abutments and mounted onto polyurethane blocks. The authors found when cemented to implant-supported zirconia abutments, additively manufactured zirconia crowns displayed fracture resistance comparable to milled ceramic crowns and there was not any significant difference between the groups. Milled zirconia crowns showed the greatest median fracture resistance (1292 ± 189 N) followed by milled lithium disilicate (1289 ± 142 N) and AM zirconia crowns (1243.5 ± 265.5 N). In all groups, a fracture line was placed at the abutment, near to the interface between the abutment and implant analog (Figure 4).

### 3.3. AM Bone Regeneration Zirconia Applications

Due to superior mechanical properties and biocompatibility, AM zirconia-based ceramics for bone generation and bone tissue engineering have recently gained much attention. Although, in contrast to the distinguished calcium phosphate-based ceramics for bone-regeneration applications, the biological activities of zirconia-based ceramics for bone-regeneration applications have not been completely investigated and only a few studies have been performed by using extrusion-based, Robocasting/Direct Ink Writing (DIW), DLP and SLS techniques [92]. 

Li et al. [81] manufactured AM zirconia scaffolds for biological engineering via direct write printing technique (DIW). A water-based zirconia ink with a solid content percentage of 70% was deposited layer by layer on the substrate using a tiny nozzle. For homogeneous grain size and a specified number of pores, sintered at 1250 °C for 4 h was the best procedure. The authors reported higher compressive strength for AM zirconia scaffolds than hydroxyapatite (HA). The proliferation of HCT116 cells was observed around the AM zirconia scaffolds by microscope. They concluded AM DIW technology can be used for manufacturing zirconia scaffolds with exact porosity control for advanced bone-tissue engineering applications.

## 4. Challenges and Future Perspectives

Additive manufacturing (AM) is developing in dentistry as a potential approach for fabricating dental restorations and other appliances. However, AM zirconia ceramic techniques are not yet well-developed, and several issues, such as difficulty in preparing raw materials, process control, and ceramic printers development must be addressed. Surface quality, dimensional accuracy, and mechanical properties must all be enhanced in order to make high-quality objects [15,32].

The feedstock for each AM technology must be given in a form that is compatible with the process. In the case of zirconia ceramics, the raw materials are currently available in the form of slurry, powder, or bulk solid, depending on the kind of AM technique [14]. The production of zirconia ceramic raw materials for AM process, generally is fraught with difficulties. Inkjet printing as a considerable technology for producing dense ceramic objects [50], it is nevertheless challenged by nozzle clogging, breakage, and thinning of the printed filament [93,94]. To address these concerns, efforts should be taken to create a stable suspension with controlled rheology and appropriate viscoelastic behavior. In the SLS process, the distribution of particle size of zirconia ceramic is crucial to the density, flowability, and shrinkage of printed objects and should be given special consideration. Furthermore, a binder with a lower melting temperature is frequently utilized to reduce crack formation during the SLS processing of ceramics [50]. During the SLA process, it is desirable to find a balance between particle size and the resulting light scattering properties. Since smaller particles increase light scattering, the penetrating depth is reduced [50].

Despite the fact that several commercially available AM systems capable of printing zirconia ceramics have been proven, there are many misalignments between present AM ceramic capabilities and application performance requirements. Surface quality is determined by the AM technology employed, processing conditions, and raw material properties, which impact the thickness of each printed layer [15]. Dimensional accuracy is essential in the creation of dental components, which must match the needs of each patient precisely, however, dimensional accuracy is affected by several factors. It is widely accepted that the accuracy in the Z-direction is lower and more difficult to improve than in the X and Y directions because it is affected by a variety of process parameters that are difficult to control such process include: spreading compaction/densification of the powder within the layers, material evaporation by laser/heat, and shrinkage during solidification [15]. Most zirconia ceramics manufactured by AM process have inferior mechanical properties compared to milled methods due to easily induced residual porosity in the printed items. To reduce porosity, various solutions have been presented. Choosing ceramic powders with an appropriate granulometric distribution, introducing dopants or a viscous liquid-forming phase, penetrating the sintered body with vitreous materials, and applying cold/hot isostatic pressure to the green body are some examples [15,22]. Shrinkage is also another concern in AM procedures since it has a substantial impact on the dimensions of the component and can lead to cracking. To minimize the influence of this phenomenon, the printing strategy must be adjusted. Increasing the amount of ceramic particles in the pastes while maintaining the rheological behavior, including particles in the mixture that can expand due to phase transformation or reactivity during sintering, and lowering the sintering temperature without sacrificing density are possible solutions to this problem [15].

The prospects for current AM methods capable of producing zirconia ceramics are thought to be in the following areas. The mechanical properties along with microstructure need to be improved to bridge the gap with milling methods. Also, to obtain the optimal mechanical properties, it is highly needed to determine the best process parameters and optimizing the process in each method. Furthermore, the limits of the AM machinery should be addressed so that zirconia parts of greater sizes, more accurate dimensions, or lower prices can be manufactured. Finally, future study into the application of AM technology should concentrate on producing biomimetic dental restorations that reflect the multi-layered as well as the complex mechanical properties of natural tooth.

## 5. Conclusions

The preliminary lab studies present different AM technologies for processing zirconia for different clinical applications mainly in restorative and implant dentistry. Each technology has some advantages, however vat polymerization including (DLP and STL) AM technology seems to be the most common in AM of zirconia for dental applications. Although in vitro studies show a comparable mechanical properties and accuracy when compared to milling and the potential of this new technology is very promising however further improvements in various areas including printer development, material development and improving the printing parameters is essential. More information regarding optical properties, biocompatibility, residual resin remnants and bonding to porcelain for layering technique is necessary before validating the technology for clinical applications. However due to its potentials and ability to produce complex geometries with different properties the application of additive manufacturing zirconia in restorative, implant and regenerative dentistry may expand quickly.

## Figures and Tables

**Table 1:** Summary of studies using AM manufactured zirconia for the fabrication of dental applications.

Author/Year	AM Technology	Application	Composition	Evaluated Parameters	Main Findings
Ebert et al., 2009 [65]	Direct Inkjet printing (DIP) (From Hewlett Packard)	Dental crown	Suspension consisted of 27 vol% zirconia powder, 55% distilled water, dispersants, and 3 mol% yttria partially stabilized zirconia powder	-Density -Sintering shrinkage -Microstructure -Mechanical properties	-Relative density: 96.9% -Isotropic shrinkage: 20 vol% -Homogeneous microstructure with some submicron-sized pores -Single bigger defects owing to clogging printing nozzles -Characteristic strength: 763 MPa -Weibull modulus: 3.5 -Fracture toughness: 6.7 ± 1.6 MPa.m1/2. -Crack-free components
Özkol et al., 2012 [70]	Direct Inkjet printing (DIP) (From HP Deskjet)	Dental bridge framework	-Ceramic ink consisted of an aqueous dispersion of 40 vol% 3Y-TZP particles (particle size 0.63 µm), Carboxylic acid-based dispersant, water, dispersants, and humectants -Supportive ink contained aqueous dispersion of sub-micron-sized thermal black type carbon black particles, alkali free carboxylic acid ester-based dispersant	-Density -Stress distributions -Maximum tensile stress -Flexural strength	-No clogging printing nozzles -Printed components had a smooth surface without any stair steps and drying or sintering cracks -Relative density: >96% -Maximum tensile stress under realistic clenching conditions: ∼340 MPa -Flexural strength (characteristic strength): ∼843 MPa -Weibull modulus: 3.6 -The defect on the supportive base was transferred to the framework structure leading a void on the top surface
Cheng et al., 2017 [82]	Stereolithography (SLA)	Dental implant	-Slurry contained zirconia-yttrium ceramic powder (EZU3YA-1) with particle size 1 µm mixed with photocurable resin at a weight ratio 13:5 -Resin binder: triethylene glycol dimethacrylate and urethane dimethacrylate, 1:3 ratio -Photoinitiator: camphorquinone Wavelength: 470 nm	-Microstructure -Hardness -Flexural strength	-Flexural strength: Green body: 20.41 ± 3.8 MPa Sintered specimens: 632.1 ± 72.5 MPa -Vickers hardness Green body: 0.12 GPa Sintered body: 14.72 GPa -Stable microstructure with no microcracks
Anssari Moin et al., 2017 [83]	Direct light processing (DLP) (from Admatec)	Root analogue implants (RAI)	-The photopolymer used was a dispersion of a commercial ceramic powder into a liquid solution of polyacrylate -Due to the patenting process, the authors could not release further details	-Dimensional accuracy	-Printed RAI had a 6.67% larger surface area and 46.38% of the printed RAI has a greater distance than 0.1 mm from the original tooth representing a volumetrically larger copy -Compared to CAD model, the printed customized implant had greater divergence for surface area change (7.14%), percentage threshold exceeding for 0.1 mm (59.33%) and 0.5 mm (4.86%).
Osman et al., 2017 [84]	Direct light processing (DLP) (From Admatec)	Dental implant	-Slurry contained commercial zirconia-yttrium ceramic powder (TZ-3YS-E) mixed with photocurable resin -3 mol% yttria -particle size 0.09 µm	-Dimensional accuracy -Density -Flexural strength -Morphology -Surface roughness --Crystallographic phase	-Dimensional accuracy: high (average deviation: 0.089 and −0.129 mm (±0.068). -Presence of several microcracks, porosities and interconnected pores. -Surface roughness Ra value: 1.59 ± 0.41 µm Rq value: 1.94 ± 0.47 µm -Flexural strength: 943 MPa
Lian et al., 2018 [23]	Stereolithography (SLA) (From Shaanxi Hengtong Intelligent Machine)	Dental bridges	Ceramic suspension: -An aqueous dispersion of 40 vol% submicron sized 3Y-TZP particles (particle size 0.2 µm) -Stir of acrylamide and methylenebisacrylamide, deionized water, and glycerol -photoinitiator liquid (PI-1173)	-Shrinkage -Density -Hardness -Surface roughness -Microstructure	-Relative density: 98.58% -Vickers hardness: 1398 HV -Isotropic shrinkage: 20 to 30 vol% -Superficial roughness: 2.06 µm -Flexural strength: 200.14 MPs -Internal defects (pores)detected
Wang et al., 2019 [85]	Stereolithography (SLA) (From 3DCeram)	Dental crown	-Photosensitive resin mixed with zirconia paste (3DMixZrO2L)	-3D trueness (In the 4 locations of the crown)	-3D printed zirconia crowns met the trueness standards.
Li et al., 2019 [86]	Stereolithography (SLA) (From Porimy)	Dental crown	Slurry of custom-made resin-based zirconia (45 vol%)	-Density, -Shrinkage, -Flexural strength -Internal and marginal adaptation	-Density: 5.83 g/cm³ Shrinkage rate was 18.1% in length (x axial), 20% in width (y axial), and 24.3% in height (z axial). -Flexural strength of 812 ± 128 MPa -Weibull modulus of 7.44 -Weibull characteristic strength: 866.7 MPa -Homogenous microstructure -Cement space of 63.40 ± 6.54 μm in the occlusal area, 135.08 ± 10.55 μm in the axial area, and 169.58 ± 18.13 μm in the marginal area which was not ideal.
Zandinejad et al., 2019 [18]	Stereolithography (SLA) (From 3DCeram)	Implant-supported AM crown	-Commercial slurry (3DMix ZrO_2_) -Zirconia paste mixed with liquid photosensitive resin -Particle size: 0.1–0.8 µm	-Fracture resistance -Mode of failure	-Fracture resistance of AM crown: 1243.5 ± 265.5 N -The fracture line was located near the interface of zirconia abutment and implant analog. -AM crowns showed comparable fracture resistance to milled restorations when cemented to zirconia abutments.
Revilla-León et al., 2020 [87]	Stereolithography (SLA) (From 3DCeram)	Dental crown	-Commercial slurry of zirconia paste (3DMix ZrO_2_ paste) mixed with liquid photosensitive resin -Particle size: 0.1–0.8 µm	-Marginal and internal discrepancies	-Higher marginal and internal discrepancies in AM groups
Ioannidis et al., 2020 [88]	lithography-based (LCM) process (similar to DLP) (From Lithoz)	Occlusal veneers	-Slurry consisted of 40–60 vol% various types of ceramic powder (3 mol% yttria stabilized zirconia particles in a purity of 99.9%) mixed with a photopolymerizable monomer (dynamic viscosity at 20 °C is 43 Pa s)	-Load-bearing capacity	-load-bearing capacities were surpassed clinically expected average bite forces -Median F initial values 1′650 N -median Fmax values 2′026 N
Wang et al., 2021 [89]	Stereolithography (SLA) (From Porimy)	Dental crown	-Commercial slurry CSL150 (Zirconia, 1,6-Hexanediol diacrylate, Pentaerythritol tetraacrylate)	-Dimensional accuracy -Clinical adaptation	-Dimensional accuracy: 65 ± 6 µm -No significant difference was found between the AM and milling zirconia crowns. -AM crowns had high dimensional accuracy and marginal adaptation within clinically acceptable limits
Li et al., 2021 [90]	Stereolithography (SLA) (From Porimy)	Dental crown	-Commercial slurry CSL 100 (47 vol% 3 mol zirconia suspension.)	-Manufacturing accuracy -Margin quality	-AM crowns indicated comparable accuracy to milled crowns. -Knife- edged crowns were susceptible to large marginal chipping. -AM crowns had rounded line angle margins and were free of small faults
Revilla-León et al., 2021 [36]	Stereolithography (SLA) (From 3DCeram)	Bar-shaped	-Commercial slurry 3DMix ZrO_2_ paste (Zirconia paste mixed with liquid photosensitive resin) -Particle size: 0.1–0.8 µm	-Flexural strength -Fracture resistance	-Mean fracture resistance value of Am specimens: 640.64 ± 81.10 N -Flexural strength of AM specimens: 320.32 ± 40.55 MPa. -Lower flexural strength for AM zirconia than milling zirconia.
Revilla-León et al., 2021 [37]	Stereolithography (SLA) (From 3DCeram)	Bar-shaped	-Commercial slurry 3DMix ZrO_2_ paste (Zirconia paste mixed with liquid photosensitive resin) -Particle size: 0.1–0.8 µm	-Manufacturing accuracy -Volumetric changes	-The 40% porosity AM zirconia had the highest manufacturing accuracy and the lowest manufacturing volume change, followed by the 20%-porosity and the 0%-porosity groups. -All the groups tested were unable to perfectly mimic the virtual design of the specimens.
Nakai et al., 2021 [38]	Stereolithography (SLA) (From 3DCeram and Lithoz)	square-shaped	-Three different commercial slurry: 1. LithaCon 3Y 230 2. 3D Mix zirconia 3. 3D Mix ATZ	-Crystallography -Microstructure -Flexural strength	-Comparable phase composition, residual porosity, and flexural strength for AM zirconia specimens with milling zirconia. -ATZ had the highest flexural strength - Lithoz showed a significantly lower biaxial flexural strength than 3D Mix zirconia -The highest Weibull modulus: 3D Mix zirconia (16.3) -The highest scale: 3D Mix ATZ (1108.8 MPa)

**Table 2:** 3D printers available for the manufacturing zirconia ceramics.

Manufacturer	3D Printer	Technology	Zirconia Grades	Composition
Lithoz	Cerafab 7500 Cerafab LabL30 Cerafab system S65	Lithographic-based ceramic manufacturing (LCM) based on a DLP technology	Lithacon 3Y 210 Lithacon 3Y 230	3 mol% yttria stabilized zirconia
3DCeram	Ceramaker 900 C3600 Ultimate	Stereolithography (SLA)	3D Mix zirconia	3 mol% yttria stabilized zirconia
			3D Mix ATZ	Alumina (20%) and Zirconia (80%)
Admatec	Admaflex 130 Admaflex 300	Direct light processing (DLP)	AdmaPrint Z130	3 mol% yttria stabilized zirconia
Porimy	CSL 150	Stereolithography (SLA)	NP	NP
Prodways	Promaker V6000	Moving Light technology, based on DLP technology	NP	NP
Exone	X1 160Pro	binder jetting	NP	NP
Lynxter	Lynxter S600D	Extrusion	NP	NP

NP: Not provided.
